# What is the Role of the Bystander Response in Radionuclide Therapies?

**DOI:** 10.3389/fonc.2013.00215

**Published:** 2013-08-19

**Authors:** Darren Brady, Joe M. O’Sullivan, Kevin M. Prise

**Affiliations:** ^1^Centre for Cancer Research and Cell Biology, Queen’s University Belfast, Belfast, UK; ^2^The Northern Ireland Cancer Centre, Belfast, UK

**Keywords:** radionuclide, radioisotope, Bystander effect, targeted alpha therapy, targeted radionuclide therapy, radiopharmaceuticals

## Abstract

Radionuclide therapy for cancer is undergoing a renaissance, with a wide range of radionuclide and clinical delivery systems currently under investigation. Dosimetry at the cellular and sub-cellular level is complex with inhomogeneity and incomplete targeting of all cells such that some tumor cells will receive little or no direct radiation energy. There is now sufficient preclinical evidence of a Bystander response which can modulate the biology of these un-irradiated cells with current research demonstrating both protective and inhibitory responses. Dependence upon fraction of irradiated cells has also been found and the presence of functional gap junctions appears to be import for several Bystander responses. The selection of either high or low LET radionuclides may be critical. While low LET radionuclides appear to have a Bystander response proportional to dose, the dose-response from high LET radionuclides are more complex. In media transfer experiments a “U” shaped response curve has been demonstrated for high LET treatments. However this “U” shaped response has not been seen with co-culture experiments and its relevance remains uncertain. For high LET treatments there is a suggestion that dose rate effects may also be important with inhibitory effects noted with 125I labelling study and a stimulatory seen with 123I labelling in one study.

## Introduction

The concept that ionizing radiation can have a biological effect upon cells which receive no direct energy deposition is now well established. Blyth and Sykes, in a recent review, have sought to standardize the definition of this Bystander response as “Radiation-induced, signal-mediated effects in un-irradiated cells within an irradiated volume” (1). These signal mediated Bystander effects include cell death, DNA damage, chromatid aberrations, genomic instability, transformation, differentiation, proliferation, gene expression, cell cycle, invasion, and radioadaptive responses. Release of signaling molecules (2) and direct intercellular communication via gap junctions are the two primary mechanisms involved (3). Cell proximity has also shown to be necessary for proliferative Bystander responses (4). Reactive oxygen and nitrogen species together with calcium and cytokines have all been implicated in Bystander signaling [see Ref. (5) a review]. Much of the evidence for Bystander response is drawn from experiments with external radiation sources with comparatively fewer reports involving radionuclides.

Clinically radionuclides have demonstrated utility in medical imaging technologies and therapeutic treatments. Radioiodine therapy with Na^131^I is well established in the treatment of differentiated thyroid cancer (6). In the treatment of lymphoma two radioimmunotherapies are commercially available ^90^Y-ibritumomab tiuxetan and ^131^I-tositumomab, both are radiolabeled anti-CD20 antibodies (7, 8). For the palliation of bone metastasis there are several therapies in use, ^89^Sr, ^153^Sm, ^186^Re, ^188^Re, ^223^Ra (9). Radioembolisation with ^90^Y microspheres has been used for the treatment of hepatocellular carcinoma. ^131^I-MIBG is indicated in the treatment of pheochromocytoma, paraganglioma, carcinoid tumor, neuroblastoma, and medullary thyroid cancer (10). Peptide receptor therapy with ^90^Y-DOTATATE/^90^Y-DOTATOC is used for the treatment of metastatic neuroendocrine tumors (11). In addition there are many more radionuclides undergoing clinical trials. There is now, however, a renewed interest in radionuclide therapy with the results of the recent ALSYMPCA trial demonstrating a survival advantage for the use of ^223^Ra in the treatment of prostate cancer bone metastasis (12) and the subsequent FDA approval of this radiopharmaceutical. The non-uniform dose deposition from radionuclide therapy and the inability to target all tumor cells provides a potential niche for Bystander responses to have a significant impact in this setting.

## Dosimetric Considerations

**Figure 1:**
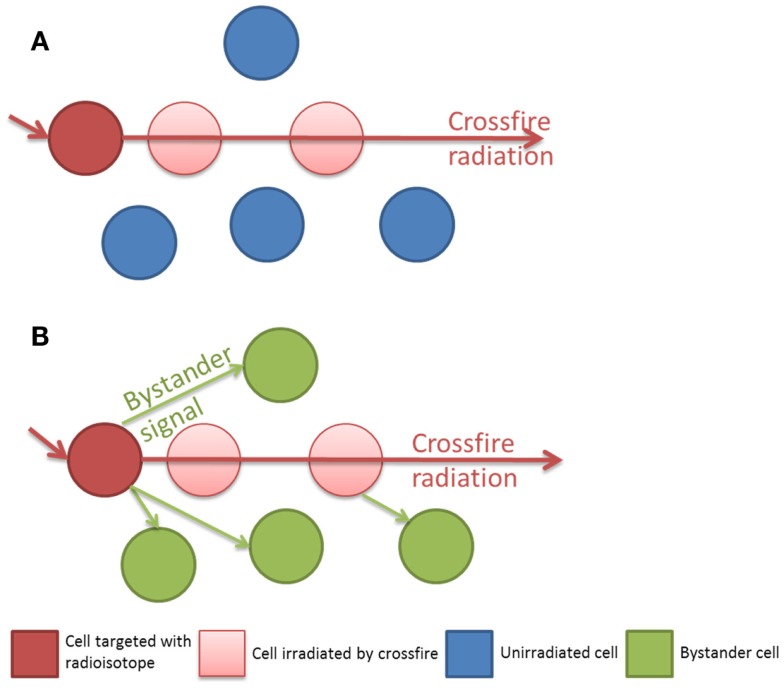
**(A)** Isolated radiation crossfire model, the biological effect to un-irradiated cells is related to the path length of the radionuclide. **(B)** Bystander and crossfire model, additional non-targeted cells receive a biological effect from Bystander signaling.

In clinical radiation therapy there are important dosimetric differences between external beam or sealed source brachytherapy and unsealed radionuclide therapy. Linear accelerator based external beam radiotherapy (EBRT) uses high energy photons (4–18 MeV) to flood a target volume, irradiating all cells within the field, including both tumor and normal tissue in the treatment field. Furthermore the total dose of EBRT is often fractionated over several weeks with each fraction typically delivered over several minutes. This is in sharp contrast to radionuclide therapy, which attempts to molecularly target dose in a tissue specific, as opposed to a volume specific, manner. The rate of dose delivery to a tumor is complex depending upon the specific activity and half-life of the radionuclide, its radiation properties (radiation types, energies, and yields), and absorption into the tumor mass, combined with the biological transit time and clearance. At a macroscopic level the dose delivered from a radionuclide appears uniform, however at the cellular and sub-cellular levels there is actually significant inhomogeneity. Many cells will not be affected directly by the radiation and the energy deposition will consist of multiple hotspots. With such non-uniform radionuclide exposures the mean absorbed dose is not the best parameter to predict biological response (13). Classically radionuclides have been chosen with radiation decay path lengths long enough to compensate for the incomplete targeting of all tumor cells but short enough to reduce the dose to surrounding normal tissue. Samarium-153 EDTMP (^153^Sm) is often used in the treatment of bone metastasis, as the effective range of its beta emission is 2–3 mm. This is long enough to deliver a dose to the adjacent tumor tissue but short enough to minimize the dose to the radiosensitive normal bone marrow. Those cells which are not directly targeted may then still receive a dose from the crossfire emanating from a neighboring targeted cell. It is in this population of cells which are un-irradiated or only sparsely irradiated by crossfire that the impact of the Bystander effect may be crucial in order to achieve sterilization of a tumor as a whole (see Figure 1). Historically the radionuclides for therapeutic use were therefore typically low LET beta emitters. Recently this paradigm has been challenged with radionuclides such as Radium-223 (^223^Ra), a high linear energy transfer (LET) alpha emitter with a short range, less than 100 μm (12).

To achieve selective tissue uptake there are several strategies to deliver radionuclides. The radionuclide can be administered as a radioactive salt such as ^89^Sr or ^233^Ra both of which are calcium mimetics and can therefore be absorbed by the dysregulated bone at the sites of skeletal metastasis. Radioiodine therapy for the treatment of thyroid cancer is also unique, as the sodium/iodide symporter predominates within thyroid tissue and selective uptake of the radionuclide is easily achieved. Such convenience is uncommon and many of the clinically available radionuclides require a carrier molecule to localize to the tissue of interest. Bisphosphonates can be used to localize to bone (^186^Re, ^188^Re, ^153^Sm) or a radioimmunotherapy approach with monoclonal antibody to epitopes such as CD20 found on malignant B lymphocytes (e.g., Ibritumomab tiuxetan ^90^Y). Radiohalogens can be incorporated into molecules such as MIBG (^131^I, ^123^I) enabling internalization into cells which express the NAT receptor such as neural crest cancers (14). Irrespective of the specificity of targeting, normal tissue often still receives a radiation dose either from crossfire in a tumor adjacent area or to the organs involved in transport and excretion of the radionuclide.

## Experimental Evidence for Bystander Response with Radionuclides

### In vitro

*In vitro* experiments to investigate the Bystander response in radionuclide therapy are more challenging than external beam experiments. There are practical issues such as ensuring there is a population of un-irradiated cells, with no radionuclide incorporation. Several studies have demonstrated a Bystander response using ^3^H (tritium) as a short range beta emitter incorporated into the DNA with thymidine (^3^H-dThd). ^3^H decays releasing low LET beta particles with a mean energy of 5.67 keV and a very short range of only 1 μm, effectively restricting the radiation dose to within the cell, with no extracellular crossfire. Early evidence of a Bystander response was demonstrated by Bishayee et al. (15) using Chinese hamster V79 cells labeled with ^3^H-dThd. These cells were mixed with varying concentrations of unlabeled cells centrifuged into multicellular clusters of about 1.6 mm in diameter and plated for a colony survival assay after 72 h. At 100% labeling of cells in the cluster colony survival depended exponentially on cluster activity. At 50% of cell labeling colony survival was lower than predicted and this was attributed to a Bystander response. Addition of the gap junction inhibitor lindane had no effect on colony survival for the 100% labeled cells but colony survival increased with dose up to a plateau for the 50% labeled cells, suggesting a role for intracellular communication in this Bystander response. Further experiments with a lower 10% labeling of cells confirmed the protective effect with lindane and also to a lesser extent the radical scavenger DMSO (16). In additional experiments utilizing ^125^I labeled iododeoxyuridine (^125^IUdR) incorporated into DNA, they labeled 100, 50, and 10% of V79 cells in a multicellular cluster. ^125^I emits predominantly Auger electrons (low energy conversion electrons and gamma rays are also emitted) depositing 99% of the dose within 0.5 μm with little extracellular crossfire. These clusters were then disaggregated and plated for colony survival. Survival of the cells in the 50 and 10% labeling groups was lower than predicted from dosimetric estimations (17). The same group performed co-culture of ^3^H-dThd labeled rat epithelial cells (WB-F344) with unlabeled cells. They used a fluorescent staining approach to discriminate the two cell populations by flow cytometry with the membrane permeant reactive tracer, carboxyfluorescein diacetate succinimidyl ester (CFDA SE). Co-culturing of labeled and unlabeled cells led to an increase in the proliferation of Bystander cells. This increase in proliferation ratios was dependent on the fraction of labeled cells present (18, 19). Persaud et al. labeled CHO cells with ^3^H-dThd and co-cultured these with unlabeled A_L_ cells in a 1:5 ratio to produce a multicellular spheroid of cells. The A_L_ cells were separated with magnetic beads and mutation analysis performed. Increased rates of mutations were found in the un-irradiated A_L_ cells together with decreased clonogenic survival. As with the previous studies the addition of lindane or DMSO was found to abrogate the mutation frequency. They similarly demonstrated a role for reactive oxygen species and gap junction intercellular communication (20, 21). Chouin and Bernardeau labeled antibodies with ^213^Bi to target two lymphoid cell lines *in vitro*. ^213^Bi is predominately a beta emitter (98% beta decay, 2% alpha decay) with a short path length of 50 μm (22). At low mean absorbed doses, cell mortality was higher than expected from modeling the probability of a radiation cell hit, suggestive of a Bystander response. Indeed it’s likely that the alpha decay of ^210^Po, a daughter product of ^213^Bi is responsible for this low dose effect. Howell et al. observed a similar phenomenon utilizing their V79 multicellular cluster model labeling with ^210^Po (23). At 1% labeling of cells a decrease in predicted clonogenic survival was noted.

Targeting normal tissue with the expectation that Bystander signaling will cause cell kill in an adjacent tumor population has many potential advantages. Mamlouk et al. labeled human lymphocytes with ^125^I labeled iododeoxyuridine (^125^IUdR) and co-cultured them with the colon adenocarcinoma cell line LS174T *in vitro*. There was decreased survival seen with the LS174T cells both in direct co-culture and with a media transfer experiment (24). To demonstrate that the Bystander response was not in fact due to crossfire from the radionuclide decay in a separate experiment the labeled cells were killed and then incubated resulting in the abrogation of the previously observed Bystander response. Akududu et al. employed a 3D carbon scaffold culture method to investigate Bystander responses in two breast cancer cell lines MCF-7 and MDA-MB-231. The cultures were pulse labeled with ^125^IUdR and 5-ethynyl-2′-deoxyuridine (EdU) to identify labeled cells by flow cytometry (25). 15% of the MCF-7 cells were labeled and 10% of the MDA cells, yet a lethal Bystander effect was only observed in the MDA-MB-231 cells.

### In vivo

Bystander responses have been demonstrated in several *in vivo* models. Xue et al. used a protocol injecting human colon LS174T adenocarcinoma cells into nude mice (26). LS174T cells were labeled with ^125^IUdR then mixed with unlabeled cells and dead cells (produced by freeze thawing and used as cell spacers). The labeled cells incorporated a lethal dose, the estimated dose received by the unlabeled cells was less than 10 cGy and the dead cells were used as spacers to ensure consistent spacing of labeled and unlabeled cells. Any delay in tumor growth could therefore be attributed to the effects on the unlabeled cells. The group observed inhibition of tumor growth at 1:1 and 1:5 labeling ratios. Metaiodobenzylguanidine (MIBG) is a molecule similar in structure to noradrenaline and as such is transported intracellularly via the Noradrenaline Transport (NAT) receptor. Neural crest tumors express this receptor and can be targeted with labeled MIBG. Clinically MIBG can be labeled with ^123^I (utilizing the gamma decay component) for imaging studies or the beta emitter ^131^I for therapeutic use. Transfection of other tumor types with the NAT gene has been explored as a potential mechanism to target radionuclide therapy within model systems. Several authors have utilized this system with ^131^I-MIBG to partially irradiate transgenic mosaic xenograft models with varying ratios of tumor cells expressing the NAT gene. While a reduction in tumor growth has been noted, beyond what would be expected from the fraction directly irradiated it’s unclear the relative proportion of cells kills by crossfire vs. a biologic Bystander effect (27–29).

## Factors that Might Affect Radionuclide Bystander Response

### The influence of LET

Micrometasis represent a challenge to deliver radionuclides at a concentration sufficient to sterilize a small volume of cells. In this scenario High LET radiation such as alpha particles have a theoretical advantage over the low LET particles used for many clinical treatments. It is estimated that between 100 and 1000 beta particle traversals are required to kill a cell, compared to between 1 and 10 alpha particle traversals (30). The probability of a beta particle inflicting a lethal lesion is therefore small at low fluences. Alpha particles however demonstrate a log-linear cell kill even at low doses, with a greater relative cell kill at low doses, compared to low LET radiation. In acknowledgment of these differences the term Targeted Alpha Therapy has been used to describe the clinical use of alpha emitting radionuclides. Boyd et al. investigated the relationship between LET and Bystander response from media transfer clonogenic experiments. Two tumor cell lines were transfected with the NAT gene and exposed to either ^123^I-MIBG, ^131^I-MIBG, ^211^At-MABG, or external gamma rays. Consistent with previous external beam Bystander studies the media from the externally irradiated cells caused a Bystander response at low doses, saturating with a maximum cell kill of 30–40% and no additional effect beyond this plateau despite higher doses. In contrast no such saturation of Bystander response was seen for the radionuclides. Following treatment with the low LET beta emitter ^131^I-MIBG a Bystander response was observed which increased in proportion to the activity added to the directly exposed cells, leading to the killing of 70–80% of the Bystander cells. The high LET emitting radionuclides ^123^I-MIBG and ^211^At-MAGB demonstrated an increasing cell kill at lower doses up to a maximum of 35–70% then the effect became less with increasing activities. This lead to a U-shaped response curve, with a high Bystander response at low activities and a lower Bystander response at higher activities. Boyd et al. investigated the relationship between LET and sub-cellular location using ^131^I (a low LET beta emitter), and ^123^I (a high LET Auger electron emitter) to label either MIBG (accumulates within the cytoplasm) or IUdR (incorporates into DNA). They irradiated HCT116 cells transformed with the NAT gene and performed media transfer to test for a Bystander response. Treatment of the cells with the low LET ^131^I caused a dose dependant decrease in clonogenic survival for both the MIGB and the IUdR experiments. However the high LET ^123^I treatment demonstrated a U-shaped response to both IUdR and MIBG treatments. Clonogenic survival decreased as the dose increased to a maximum effect at 4 MBq mL^1^ after which at higher doses the effect became proportionally less. They concluded that radiopharmaceutical-induced Bystander effects may depend on LET of the decay particles but are independent of the site of intracellular concentration of the radionuclide (31). Paillas et al. demonstrated a Bystander response in a media transfer experiment with ^125^I labeled monoclonal antibodies targeting CEA (non-internalizing, membrane destined) and HER2 (internalizing, cytoplasm destined) on HCT116 cells (32). Increased numbers of gamma-H2AX foci, indicative of double strand DNA breaks, were observed in recipient cells incubated in media from both the CEA and HER2 exposed populations suggesting that high LET Auger electrons can induce a Bystander response from both membrane and cytoplasmic targeting. It also confirms microbeam studies indicating sensitive sites for radiation exposure outside the cell nucleus impacting on both direct (33) and Bystander responses (34). Kishikawa et al. compared the effects of two high LET labeling strategies with ^123^I and ^125^I in an *in vivo* model. They reported two opposing effects, an inhibitory response for the ^125^I labeled study and a stimulatory response for ^123^I labeled study. These observations were also confirmed *in vitro*. While both ^123^I and ^125^I have similar emission spectra there are marked differences in radionuclide half-life (^123^I *t*_1/2_ = 13.3 h and ^125^I *t*_1/2_ = 60.5 days). For this high LET model dose rate effects may therefore be important (35).

### Clinical relevance

Investigation of a Bystander response within a clinical setting would be exceptionally challenging. For both external beam and radionuclide approaches, any Bystander response will be in addition to the direct effects of radiation exposure. It is likely that designing a clinical trial to specifically test the extent of an effect could only be done under conditions were a complimentary molecularly targeted approach to either enhance tumor cell kill or protect normal tissue by Bystander signaling would be feasible. There is renewed interest in radionuclide therapies, once seen as a palliative tool for bone metastasis or treating rare tumors such as thyroid cancer, they are now being investigated for a large variety of tumor types. The basic principle that not all tumor cells can be targeted remains, however what has changed is our approach to those untargeted cells. Together with the radiation crossfire effect, the contribution of Bystander signaling must also be considered. Incorporation of a Bystander effects model could have profound implications on the modern design and clinical delivery of such radionuclides. Understanding Bystander signaling mechanisms may allow us to more optimally target tumors and protect surrounding normal tissues (5). The relative contributions of radiation crossfire and Bystander effect are particularly important to many of the clinically available therapies. The ratio of cells that need to be targeted to achieve a Bystander effect is also important; establishing thresholds for Bystander activation may set the bar for a therapeutic benefit. It is likely that individual tumor characteristics are important as demonstrated by the presence and absence of Bystander responses in two different breast cancer cell lines (25). Indeed whether this response is protective (advantageous for normal tissue) or inhibitory (advantageous for tumor sterilization) needs to be established for each radionuclide, delivery system, dose, and tumor combination. The mechanism of the Bystander effect for high LET therapies also requires further investigation, with some *ex vivo* evidence suggesting a favorable dose at which maximum Bystander effect is achieved; which if true *in vivo* has important implications for the fractionation and dosing of such treatments.

There are important implications for clinical imaging technologies utilizing ^123^I such as ^123^I-MIBG which decays by electron capture with Auger electron emission but clinically the small gamma component is utilized to image tumors deep within a patient. Previously it was thought that any direct DNA damage would be small, caused by a few higher energy Auger electrons or conversion electrons with most of the Auger electrons of such short range to be of no threat to the nucleus. The Bystander effects observed from such decays suggest that an indirect mechanism can cause DNA damage which could result in an underestimation of secondary cancer risk from the Linear no Threshold model (36). Modulation of these Bystander responses will also be a therapeutic goal. Quenching it may, in theory at least, reduce the risk of secondary cancers, while enhancing the Bystander response could result in therapeutic gain for cancer treatment.

## Conflict of Interest Statement

The authors declare that the research was conducted in the absence of any commercial or financial relationships that could be construed as a potential conflict of interest.
